# Effect of splitting the sub-lethal dose of glyphosate on plant growth shikimate pathway-related metabolites and antioxidant status in faba beans

**DOI:** 10.1038/s41598-025-87799-7

**Published:** 2025-03-28

**Authors:** Ragab El-Mergawi, Mahmoud El-Dabaa, Fathia Elkhawaga

**Affiliations:** Botany Department, Agricultural and Biology Institute, National Research Centre, El-Tahrir Street, Dokki, 1222 Cairo Egypt

**Keywords:** Glyphosate, Splitting treatments, Faba bean varieties, Phenolics, Aromatic amino acids, Antioxidants, Physiology, Plant sciences

## Abstract

Glyphosate exerts its herbicidal activity by inhibiting the shikimate pathway, the main source of many primary and secondary metabolites. Application of a low dose of glyphosate to faba bean plants was effective in controlling *Orobanche crenata* infestation, but some toxic effects on host plants can occur. Splitting the low glyphosate dose can serve as a mitigation strategy to reduce the host toxicity. Under parasitic-free conditions, a greenhouse experiment was conducted during two seasons to study the effect of dividing the recommended glyphosate dose (170 g a.i. ha^-1^) into two-five sprays on the growth, shikimate-related metabolites, and antioxidant status of three faba bean varieties. After 40 days, splitting treatments tended to cause cumulative inhibition effects on the growth and productivity traits of faba beans depending on the tested varieties, seasons, and the number of sprays applied. The maximum reduction effect was noticed for twice- sprayed treatment in the first season and for five-sprayed treatment in the second one. The cumulative effect of splitting glyphosate treatments on the shikimate pathway metabolites and the antioxidant status was measured after a week of spraying. Splitting treatments induced great increases in shikimic acid and phenylalanine contents compared with control. These treatments continued to exert their oxidative stress on faba bean plants by reducing antioxidant activity and antioxidant compounds such as total phenolics, flavonoids, and the detected phenolic acids *(p*-hydroxybenzoic, syringic, vanillic, coumaric, and ferulic). A significant increase in the activities of antioxidant enzymes (superoxide dismutase, peroxidase, and polyphenol oxidase) was recorded for all splitting treatments.

## Introduction

Faba bean (*Vicia faba* L.) is a major part of the Egyptian diet. Faba beans are considered the main host of the* Orobanche crenata* holoparasite, which can decrease the host yield by up to 80%. One of the most effective methods for controlling the* Orobanche* parasite is the spray of low doses of glyphosate on faba bean plants that show relative tolerance to this low dose^1^. This herbicide moves through the host phloem to attach *Orobanche* and then inhibits parasite growth. To control *O*.* crenata* infestation, it is recommended to spray glyphosate herbicide on faba bean leaves at 85 g a.i. ha^-1^ twice at the early flowering stage, 21day intervals^2^. Application of glyphosate at doses that effectively controlled *Orobanche* caused phytotoxicity and reduced the host (faba bean and sunflower) yield^3–5^.

Glyphosate [*N*-(phosphonomethyl) glycine] is a non-selective herbicide; its phytotoxicity is attributed to inhibiting 5-enolpyruvylshikimate-3 phosphate synthase (EC 2.5.1.19, EPSPS), an enzyme in the shikimate pathway that is required for the synthesis of the aromatic amino acids (AAAs), phenylalanine (Phe), tyrosine (Tyr), and tryptophan (Trp)^6^. These three AAAs are precursors to protein and a wide variety of secondary plant products. The enzymatic deamination of Phe and Tyr via phenylalanine ammonia lyase (PAL) or tyrosine ammonia lyase (TAL), respectively represents the largest group of secondary metabolites in plants, including flavonoids, phenolic acids, tannins, lignans, and coumarins^7^. The effect of low glyphosate doses on metabolites related to the shikimic acid pathway, including phenolic compounds, was observed previously^2^. A rapid accumulation of phenolic compounds such as kaempferol, gallic acid, myricetin, *p*-hydroxybenzoic acid, quercetin, salicylic acid, t-cinnamic acid, catechin, benzoic acid, ferulic acid, protocatechuic acid, veratric acid, and vanillic acid in *Codonopsis lanceolata* plants was observed after glyphosate treatments^8^.

Oxidative stress in plants exposed to glyphosate due to the increase in the reactive oxygen species (ROS) was reported^9^. Plants utilize a well-organized antioxidative defense system to scavenge ROS. The antioxidant system includes enzymatic and non-enzymatic low-molecular-weight compounds that are capable together to prevent cell oxidative damage via inhibiting ROS formation. The antioxidant enzymes include superoxide dismutase, SOD; peroxidase, POD; polyphenol oxidase, PPO; catalase, CAT; ascorbic peroxidase, APX; glutathione peroxidase, GR; and glutathione-s-transferase, GST, whereas the non-enzymatic molecules include phenolics, ascorbic acid, glutathione, tocopherol, and many other compounds^8,10^. Glyphosate application showed to induce diverse effects on enzymatic and non-enzymatic antioxidants in different plant species^11,12^.

Although, for controlling *Orobanche* parasites, glyphosate was sprayed on infected and non-infected plants, there is a lack of information in the literature on the effect of the low herbicide dose on host plants under parasite-free conditions. Splitting the recommended glyphosate dose in order to control the *Orobanche* parasite can reduce glyphosate-induced phytotoxicity to host plants while still sustaining the toxic effect on the attached parasite^13^. Therefore, splitting the low glyphosate dose can serve as a mitigation strategy to reduce the toxicity that occurs to healthy and infected host plants. Thus, the current study is conducted under parasite-free conditions to assess the possibility of dividing the recommended glyphosate dose (170 g a.i. ha^-1^) into 2–5 sprays to reduce its negative effect on plant growth, shikimic acid pathway-related metabolites, and antioxidant status in three faba bean varieties.

## Materials and methods

### Plant materials and treatments application

Two pot experiments were conducted on November 15 during two successive seasons at the National Research Centre, Giza, Egypt. The experiment was repeated during the 2021/2022 and 2022/2023 seasons to avoid the climatic effects on the response of plants to tested treatments. Faba bean (*Vicia faba*) seeds of the three varieties, Giza 716, Nubaria 4, and Nubaria 5, were purchased from the Agricultural Research Centre, Egypt. Five seeds were sown in each pot (30-cm-diameter) that was filled with 5 kg of sandy soil (74.1% sand, 19.2% silt, and 6.7% clay) with pH 8.0, organic matter 1.45%, electrical conductivity 1.24 mmohs cm^-1^, CaCO_3_ 1.16%, total N 0.04%, total P 0.038% and total K 0.03%. At sowing, commercial rhizobia and 8 g of superphosphate (15% P_2_O_5_) were incorporated into the top 30 mm of the soil of each pot. Plants were subjected to similar irrigation practices and supplied with nitrogen (ammonium sulfate, 21% N, 6 g/pot) three times at 4, 6, and 8 weeks after sowing. The experiment was a factorial design with a number of glyphosate sprays (5 treatments) and three local faba bean varieties (3 varieties) in a randomized block design with six replicates. Glyphosate herbicide (Roundup, 360 g a.i. L^-1^, Monsanto, USA) at the recommended dose of 170 a.i. g ha^-1^ (2.13 mM) was divided into 2–5 sprays. The rates of different spray treatments and their application times are shown in Table 1. It is recommended to spray glyphosate herbicide on faba bean leaves at the early flowering stage to control *Orobanche* infestation. Hence, a sequential application of all splitting treatments was conducted at the beginning of the flowering stage (December 31) within a period of one month. An equal amount of the examined glyphosate solution was sprayed on the surfaces of plants in each pot by using an Epoca sprayer (Italy). At 40 days after the end of all splitting treatments (117 days after planting, DAP), plant samples from the two examined seasons were collected. Then the cumulative effect of different glyphosate splitting treatments on growth and productivity traits was estimated, including plant fresh weight (g), plant dry weight (g), and the number and weight (g) of pods per plant. The 4^th^ leaf of the faba bean plant sample of the second season was collected after a week from the end of all splitting treatments (February 8, 2022, 84 DAP) and fast-cleaned with distilled water to remove glyphosate. Then fresh samples of each replicate were frozen at − 80 °C until the antioxidant enzyme assays. The other leaf samples that were taken from each replicate were dried in an oven at 70 °C, ground with an analytic mill, and stored at -20 °C prior to analysis.

**Table 1:** Glyphosate application methods.

Number of sprays	Glyphosate dose		Days interval	Spraying period DAP
	g a.i. ha^-1^	mM		
Un-sprayed	0.0	0.0	––	–––
2 sprays	85.0	1.065	20	58–77
3 Sprays	56.7	0.704	13	52–77
4 Sprays	42.5	0.533	9	49–77
5 Sprays	34.0	0.426	7	46–77

### Determination of total phenolics

The 70% acetone extracts of the dried leaves were performed to determine the total phenolic contents by using the Folin-Ciocalteu reagent^14^. In detail, one 100 μL of extract was mixed with 1.9 ml of deionized water and 0.25 ml of Folin reagent and then mixed vigorously for 6 min. After addition of 2.5 ml of sodium carbonate (70 g L^-1^), the mixture was allowed to stand at room temperature for 1 h. The blue color formed was measured at 765 nm using a spectrophotometer (Shimadzu 240, Japan). Using gallic acid as a standard, the results were expressed as mg gallic acid g^-1^ dry sample (mg GAE g^-1^ DW).

### Determination of antioxidant activity

Antioxidant capacity was measured by using a 1,1-diphenyl-2-picryl-hydrazil (DPPH) reagent^15^. In detail, 0.75 ml of 70% acetone extract was added to 1.5 ml of DPPH solution (0.02 mg/ml methanol) and then stirred. The residual color was estimated after 5 min of reaction by using a spectrophotometer (Shimadzu 240, Japan) at a wavelength of 517 nm and compared with the color of the blank control. The antioxidant activity was calculated depending on the Trolox standard curve, and the obtained results were expressed as μmol Trolox g^-1^ dry sample.

### High-Performance Liquid Chromatography (HPLC) analysis of free AAAs

The AAAs in dried samples was extracted by acidified water (pH 2.5)^16^. The extracts were centrifuged at 4500 g for ten min and the supernatants were filtered using a 45 μm nylon filter and subjected to analysis by High Performance Liquid Chromatography (HPLC), LC-10 AD, Shimadzu, Japan. The HPLC was attached with a Luna RP-C18 (2) column (250 × 4.6 mm i.d., 5 μm, Phenomenx). Using phosphate buffer (pH 6.5) in methanol (90:10, v/v) at a flow rate of 0.9 ml/min as a mobile phase and Uv detector at 220 nm. The retention times of the three standards AAAs, Tyr, and Trp (Sigma-Aldrich, Sweden) are 6.08, 9.32, and 11.59 min, respectively.

### HPLC analysis of shikimic acid

The acidified water extract of leaves was used for shikimic acid determination by HPLC at the former mentioned conditions. The mobile phase consists of 5 mM ammonium acetate and methanol (72:28, v/v) at a flow rate 0.8 ml/min^17^. The retention time of the standard shikimic acid (Sigma-Aldrich, Sweden) is 3.6 min.

### HPLC analysis of phenolic acids

Free phenolic acids in dried leaves were extracted according to McKeehen et al.^18^. In detail, about 20 ml of 4 M NaOH was added to 500 mg of sample in a 50 ml Pyrex tube, shaken in the dark with a shaker at 150 rpm for 2 h. The suspension was acidified with cold 6 N HCl until pH 2 and centrifuged at 3000 g. Phenolic acids in the supernatant were extracted with ethyl acetate (3 × 50 ml) with a separatory funnel. The ethyl acetate fraction was dried by sodium sulfate anhydrous, filtered, and evaporated with a rotary evaporator (at 40 °C). The residue was dissolved with 2.5 ml of HPLC-grade methanol and filtered with a syringe PTFE filter of 0.2 μm pore size before analysis. Chromatographic analysis was performed on a Shimadazu LC-10 AD apparatus with a Kromasil RP-C18 column (250 × 4.6 mm i. d., 5 μm). A mixture of acetate buffer and acetonitrile (9:1, v/v) was used as the mobile phase. The buffer solution was prepared by adding 20 ml acetic acid to 6.35 g sodium acetate in deionized water and diluting the solution to 1 L with water. The retention times of the standards protocatechuic, *p*-hydroxybenzoic, vanillic, syringic, coumaric, and ferulic are 5.1, 8.0, 9.4, 10.1, 19.37, and 24.8 min, respectively.

### Enzyme extraction and assay

For the antioxidant enzyme analysis 0.5 g of fresh leaf was homogenized in 5 ml of ice-cold potassium phosphate buffer (pH = 7) containing 0.1 mM of EDTA. The homogenate was centrifuged (4 °C) for 15 min at 45,000 g, and the supernatant was used for antioxidant enzyme assay.

The peroxidase (PO) assay system contained 0.32 ml of 5% pyrogallol, 0.16 ml of 147 mM-H_2_O_2_, 0.32 ml of phosphate buffer pH 6.5, 2.1 ml of H_2_O, and 0.1 ml of enzyme extract. The change in absorbance after incubation for 5 min was determined by a spectrophotometer (Shimadazu 240, Japan) at 420 nm^19^. The polyphenol oxidase assay (PPO) mixture comprised 2.8 ml potassium phosphate buffer (100 mM, pH 6.0) containing 1.3 mM pyrogallol and 100 μl of enzyme extract. The increase in absorbance was monitored during 3 min at 430 nm^19^. The superoxide dismutase (SOD) assay mixture (3 ml) consisted of 50 mM sodium phosphate buffer (pH 7.6), 0.1 mM EDTA, 50 mM sodium carbonate, 12 mM L-methionine, 50 μM NBT, 10 μM riboflavin, and 100 μl of crude extract^20^. A blank control was performed without crude enzyme. After 15 min of illumination at 4000 lx, the color was measured at 560 nm. One unit of enzyme activity was defined as that amount of enzyme that reduced the absorbance to 50% as compared with the blank control.

### Statistical analysis

Data of five replications were statistically calculated by analysis of variance (ANOVA) using Graphics Plus Version 5.1 software. Duncan’s Multiple Range Test at 5% probability was used to compare the mean values.

## Results

### Growth and productivity traits

#### The mean effect of the number of sprays on the growth and productivity traits

**Fig. 1:**
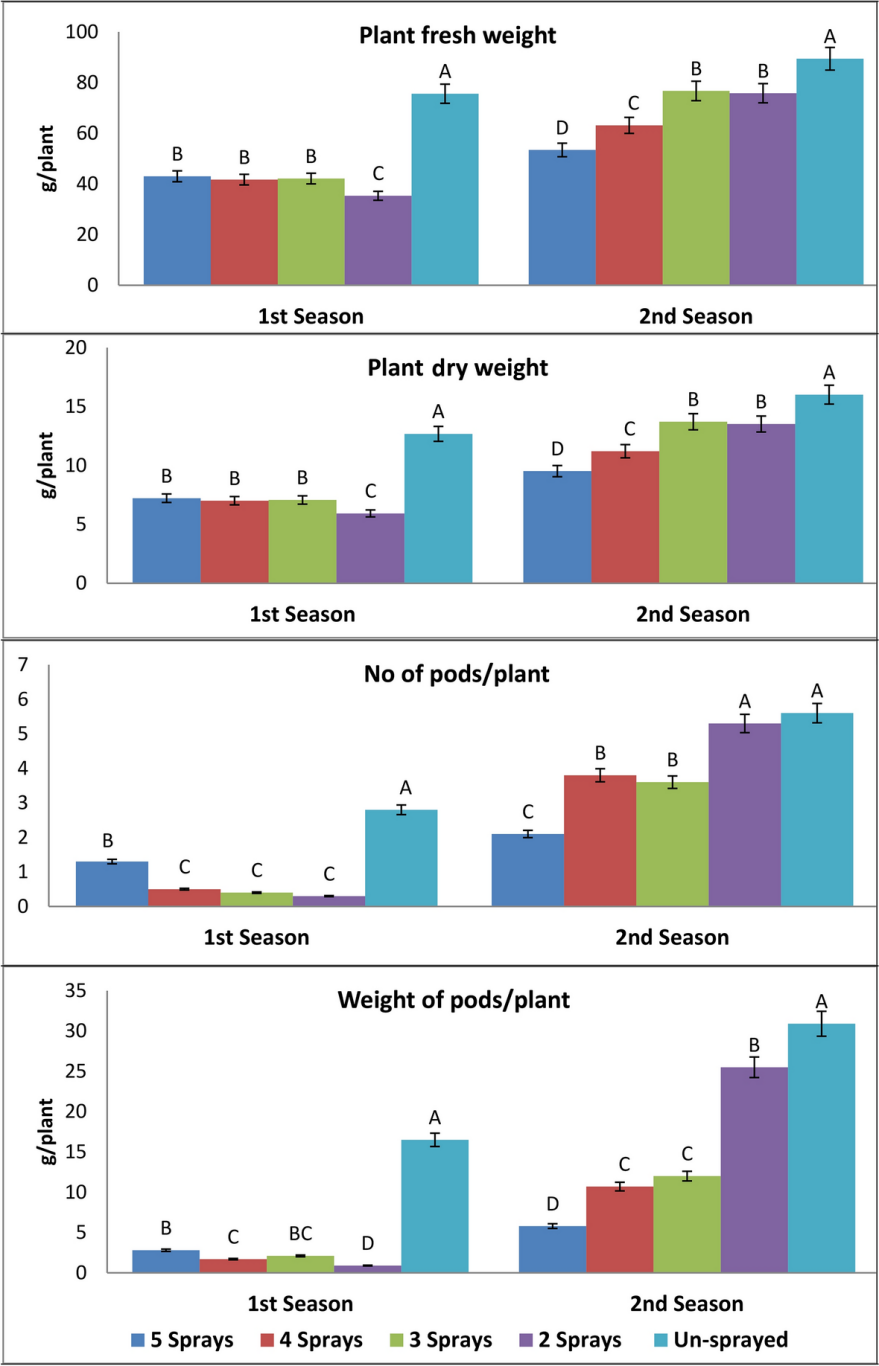
Effect of splitting sub-lethal dose of glyphosate on weight and pod yield/plant at two seasons, 40 days after treatments (mean of three varieties). The same letters are not significantly different (p < o.o5).

All glyphosate split treatments reduced plant fresh and dry biomass between 43 and 53% in the first season and between 14 and 40% in the second one, relative to corresponding unsprayed plants (Fig. 1). Meanwhile, the greatest decrease in plant biomass was recoded for two sprays in the first season and for five sprays in the second one. Moreover, the number and weight of pods per plant showed a remarkable decrease as affected by different glyphosate splitting treatments when compared with those of the corresponding control plants (Fig. 1). Splitting treatments decreased the number of pods between 53.6% (five sprays) and 89.3% (two sprays) in the first season, compared with 4.5% (two sprays) and 63.1% (five sprays) in the second one. Whereas the effect of the number of sprays applied on the weight of pods varied in the two seasons, a maximum decrease in pod weight was observed for two sprays (94.8%) in the first season and for five sprays (81.2%) in the second one.

#### Mean growth and productivity traits of three faba bean varieties

Growth and productivity traits of three faba bean varieties varied in their response to glyphosate treatments in the two examined seasons (Table 2). In the first season, the maximum value of the growth and yield parameters exhibited Nubaria 4 plants, but the lowest values tended to be obtained by Giza 716 plants. Meanwhile, in the second season, Giza 716 plants produced the highest number and weight of pods per plant.

**Table 2:** The interaction effects between splitting glyphosate treatments and faba bean varieties on weight and pod yield/plant in the two examined seasons, 40 days after glyphosate treatments.

Variety	Criteria	Fresh weight (g/plant)		Dry weight (g/plant)		No of pods/plant		Weight of pods/plant (g)	
	Sprays number	1^st^ Season	2^nd^ Season	1^st^ Season	2^nd^ Season	1^st^ Season	2^nd^ Season	1^st^ Season	2^nd^ Season
Giza 716	Five	34.3	55.0	5.78	10.5	1.3	3.9	1.0	11.9
	Four	34.7	51.3	5.8	9.8	0.0	3.9	0.0	13.2
	Three	30.7	73.7	5.2	14.1	0.0	5.4	0.0	19.2
	Two	38.3	65.7	6.4	12.6	0.2	5.5	0.7	33.0
	Control	64.0	88.3	10.8	16.9	1.7	4.7	8.9	33.5
	Mean	40.4	66.8	6.8	12.8	0.6	4.7	2.1	22.2
Nubaria 4	Five	46.3	41.3	7.8	7.4	1.5	0.5	4.7	0.6
	Four	54.3	64.7	9.1	11.7	0.4	4.2	1.5	13.0
	Three	48.7	79.3	8.2	14.3	0.5	2.7	3.5	9.6
	Two	33.0	81.0	5.5	14.6	0.5	5.9	1.4	22. 9
	Control	93.3	92.3	15.7	16.6	3.4	5.0	19.9	34.0
	Mean	55.1	71.7	9.3	12.9	1.3	3.7	6.2	16.0
Nubaria 5	Five	48.3	64.0	8.1	10.6	1.2	1.9	2.7	4.9
	Four	36.0	73.3	6.1	12.2	1.0	3.4	3.7	5.9
	Three	47.0	77.0	7.9	12.8	0.7	2.8	2.7	7.2
	Two	34.7	80.7	5.8	13.4	0.4	4.5	0.5	20.5
	Control	69.3	87.7	11.6	14.6	3.4	7.0	20.7	25.0
	Mean	47.1	76.5	7.9	12.7	1.3	3.9	6.1	12.8
LSD_5%_ V V × S		11.7 7.9	11.0 6.1	2.0 4.9	2.1 1.2	0.6 0.5	1.8 1.3	3.8 1.3	4.9 3.4

#### Interaction effects between glyphosate treatments and faba bean varieties on the growth and productivity traits

In the first season, Giza 716 plants that were treated with different glyphosate treatments produced significant decreases in the plant fresh weight, plant dry weight, number of pods and weight of pods per plant as compared with those of un-sprayed plants (Table 2). This variety tended to exhibit the highest decrease in these growth parameters for the three and four spray treatments. Treating Nubaria 4 and Nubaria 5 plants with different numbers of sprays achieved significant decreases in their fresh and dry weight as well as the number and weight of pods per plant, as compared with glyphosate-unsprayed plants. Whereas, the great reduction effect on growth and productivity traits of these varieties was recorded for two sprays treatment.

In the second season, it can be observed that glyphosate-treated plants tend to reduce fresh weights, dry weights, and the number and weight of pods per plant for the three tested varieties in most cases (Table 2). Among the three tested varieties, foliar sprays of low glyphosate dose as five sprays tended to produce the greatest decrease in fresh weight, dry weight, number, and weight of pods per plant when compared with control.

### Shikimate accumulation

#### Effect of the number of sprays on the shikimate accumulation in the three faba bean varieties

A significant increase in shikimate content was observed in the three tested varieties as affected by all numbers of sprays applied, compared with control unsprayed ones (Table 3). The enhancement effect of glyphosate treatments on shikimate was varied between the tested varieties, and the Nubaria 5 plants had more shikimate than those of either Giza 716 or Nubaria 4 plants (Table 3).

**Table 3:** Effect of splitting sub-lethal dose of glyphosate on the level of shikimic acid in the leaves of the three faba bean varieties.

Sprays No	5 Sprays	4 Sprays	3 Sprays	2 Sprays	Control	Mean
Varieties						
Giza 716	2016^A^	1718^B^	1923^AB^	1223^C^	440^D^	1464^b^
Nubaria 4	1356^B^	1415^AB^	1551^A^	1861^C^	430^D^	1163^c^
Nubaria 3	2478^A^	1832^B^	2547^A^	923^C^	692^D^	1694^a^

The same capital letters in a row are not significantly different (p < o.o5).

The same small letters in a column are not significantly different (p < o.o5).

#### Mean effects of the number of sprays on shikimate content

**Fig. 2:**
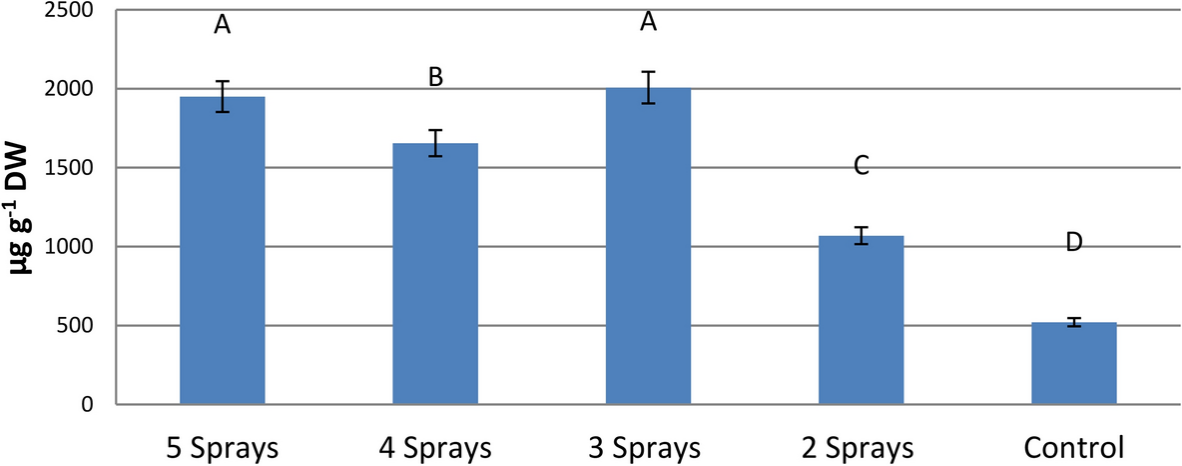
Mean effects of splitting dose of glyphosate on the levels of the shikimic acid in faba bean leaves (mean values of three varieties), 7 days after treatments. The same capital letters are not significantly different (p < o.o5).

A great increase in shikimate contents was observed for all number of sprays applied when compared with unsprayed ones (Fig. 2). Twice-spray treatment showed the least increase (105%), whereas the greatest increase was observed for 3, 4, and 5 sprays to be 285%, 218%, and 274%, respectively, as compared with those of unsprayed leaves.

### Free aromatic amino acids (AAAs)

#### Effect of the number of sprays on the free AAA contents in the three faba bean varieties

Free AAAs in the three tested varieties showed great variations in their response to different numbers of sprays applied (Table 4a,b,c). The three tested varieties did not show a unique response to the number of sprays applied, but glyphosate-treated plants of the three tested varieties tended to accumulate more Phe, Trp, and with less extended Tyr contents than those accumulated in unsprayed plants. Depending on the mean value of each variety, the three tested varieties did not show significant variations in their Tyr or Trp contents, whereas Nubaria 4 plants had the highest Phe content.

**Table 4:** Effect of splitting sub-lethal dose of glyphosate on the free aromatic amino acids Phe, Tyr and Trp in leaves (µg g^-1^ DW) of three faba bean varieties, 7 days after treatments.

Varieties									
Sprays	G 716	Nub 4	Nub 5	G 716	Nub 4	Nub 5	G 716	Nub 4	Nub 5
AAAs	(a) Phe			(b) Tyr			(C) Trp		
5 Sprays	1074^B^	1276^B^	794^B^	428^A^	424^A^	594^A^	72^AB^	122^A^	76^A^
4 Sprays	1134^A^	1160^D^	805^B^	438^A^	438^A^	321^C^	85^A^	99^AB^	73^A^
3 Sprays	1019^B^	1303^B^	988^A^	413^AB^	453^A^	464^B^	71^AB^	62^B^	71^A^
2 Sprays	1171^A^	1506^A^	537^C^	366^B^	460^A^	308^C^	82^A^	73^B^	62^AB^
Control	1058^B^	1221^C^	495^C^	385^B^	427^A^	397^BC^	50^B^	36^C^	57^B^
Mean	1091^b^	1293^a^	724^c^	406^a^	440^a^	417^a^	72^a^	78^a^	68^a^

G 716 = Giza 716, Nub = Nubaria. The same capital letters in a column are not significantly different (p < o.o5). The same small letters in a row are not significantly different (p < o.o5).

#### Mean effects of the number of sprays on free AAAs composition

**Fig. 3:**
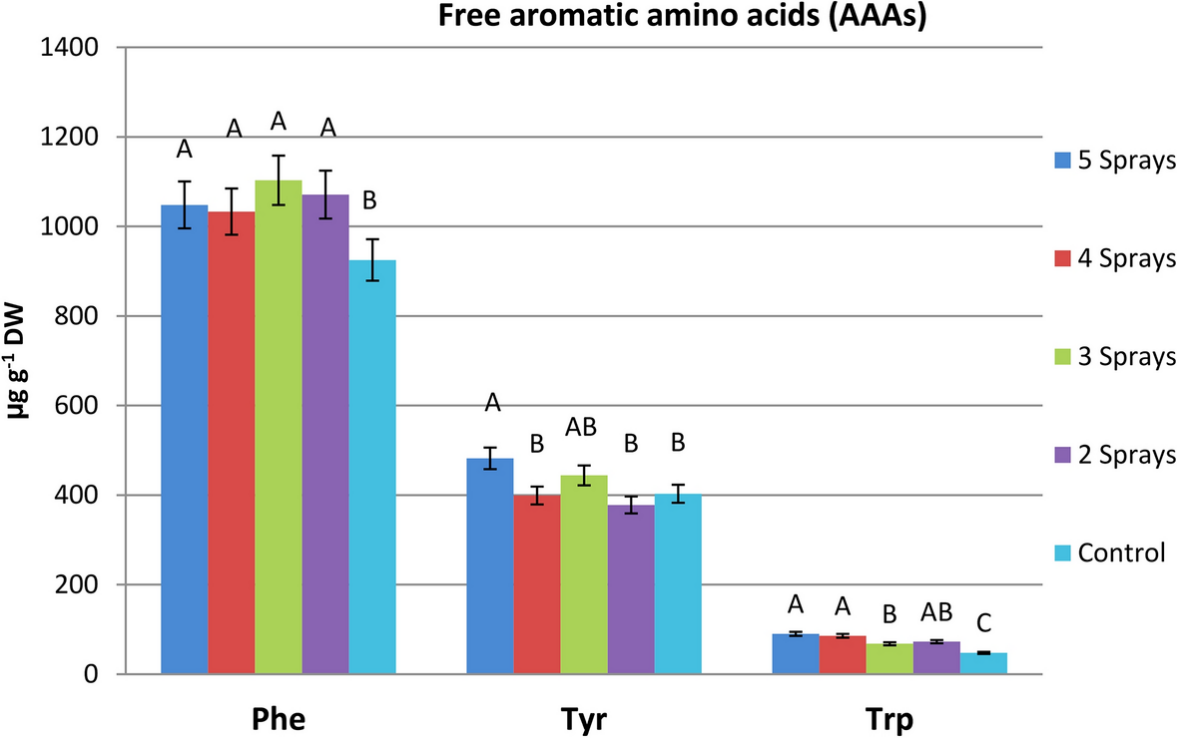
Mean effects of splitting sub-lethal dose of glyphosate on the levels of the free aromatic amino acids, Phe, Tyr, and Trp in faba bean leaves (mean value of three varieties), 7 days after treatments. *The same capital letters are not significantly different (p* < *o.o5).*

All splitting treatments tended to produce increases in the levels of the AAAs as compared with those of unsprayed plants (Fig. 3). Increases in Phe and Trp in all glyphosate-treated plants reached the level of significance, but the significant increase in Trp content was observed only for the three and five spray treatments. Using the glyphosate dose as two sprays continued to exert significant increases in the three AAAs when compared with those of unsprayed plants. This treatment caused increases in Phe, Tyr, and Trp concentrations of 13.3%, 19.6%, and 89.3%, respectively.

### Total phenolic contents

#### Effect of the number of sprays on the total phenolic and total flavonoid contents in the three faba bean varieties

Plants of the three tested varieties showed remarkable reductions in their phenolic contents as affected by all spray treatments when compared with the corresponding un-sprayed ones (Table 5a). The tested varieties showed the lowest phenolic value for four spray treatment. Depending on the mean value, Giza 716 plants accumulated more phenolics than Nubaria 4 and Nubaria 5 plants.

**Table 5:** Effect of splitting sub-lethal dose of glyphosate on the level of the total phenolics and total flavonoids in the leaves of the three faba bean varieties.

Varieties	Giza 716	Nubaria 4	Nubaria 5	Giza 716	Nubaria 4	Nubaria 5
Sprays	(a) Total phenolics (mg GAE g^-1^)			(b) Total flavonoids (mg Rutin g^-1^)		
5 Sprays	49.4^BC^	48.9^A^	51.2^B^	12.34^D^	12.53^C^	14.28^B^
4 Sprays	46.3^C^	44.1^A^	39.7^D^	14.44^B^	11.68^C^	14. 49^B^
3 Sprays	58.2^A^	46.2^A^	45.8^C^	12.92^C^	15.40^B^	14.28^B^
2 Sprays	52.0^B^	45.5^A^	45.7^C^	14.84^AB^	15.04^B^	17.72^A^
Control	60.0^A^	47.5^A^	55.2^A^	15.45^A^	17.21^A^	16.83^A^
Mean	53.2^a^	46.4^b^	47.5^b^	14.00^b^	14.17^b^	15.52^a^

The same capital letters in a row are not significantly different (p < o.o5).

The same small letters in a column are not significantly different (p < o.o5).

With one exception, a significant decrease in flavonoids content from all spraying treatments was observed for all tested varieties when compared with the corresponding control (Table 5b). Among the tested varieties, the number of sprays varied in their effects on flavonoid contents, but two sprays treatment continued to produce the lesser reduction effect. While the greatest reduction was obtained by 5 sprays for Giza 716, by 4 sprays for Nubaria 4, and by 3–5 sprays for Nubaria 5. Meanwhile, the Nubaria 5 plants accumulated more flavonoids than the other two varieties.

#### Mean effects of the number of sprays on total phenolics and total flavonoids

**Fig. 4:**
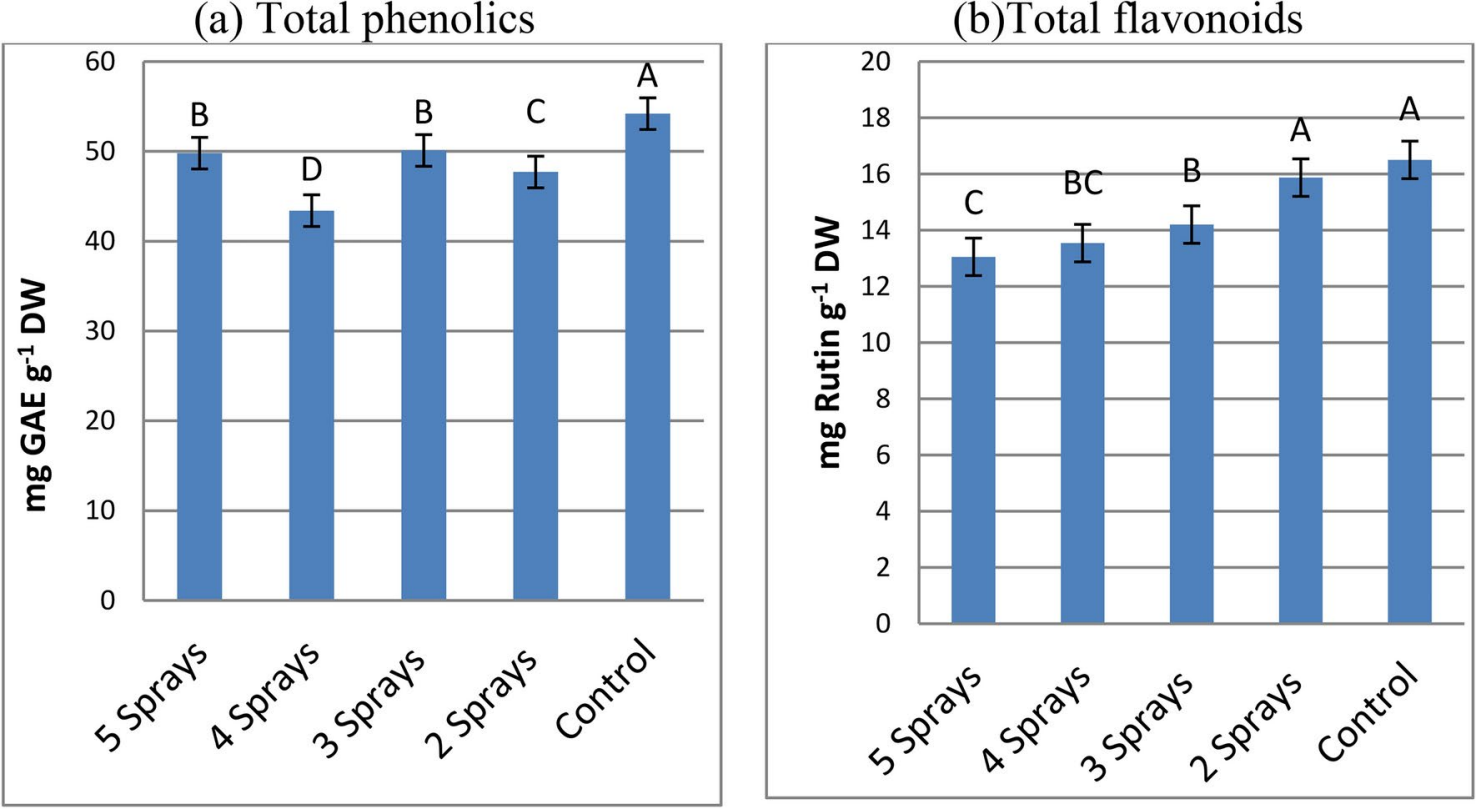
Mean effects of splitting sub-lethal dose of glyphosate on the levels of the total phenolics and total flavonoids in faba bean leaves (mean value of three varieties), 7 days after treatments. The same capital letters are not significantly different (p < o.o5).

A significant decrease in phenolic content was observed for all spray treatments as compared with un-sprayed ones (Fig. 4a). Moreover, flavonoid content was diminished by most spraying treatments, relative to control (Fig. 4b). A gradual decrease in flavonoid contents was observed with an increasing number of sprays; consequently, the maximum reduction was obtained by a 5 spray treatment (20.9%).

### Phenolic acid compositions

Four benzoic-derived phenolic acids (*p*-hydroxybenzoic, vanillic, protocatechuic, and syringic) and two cinnamic-derived phenolic acids (coumaric and ferulic) were detected in the leaves of the three faba bean varieties with different concentrations. *Effect of the number of sprays on the phenolic acid composition in the three faba bean varieties.*

(i) Benzoic-derived phenolic acid

HPLC analysis revealed that* p*-hydroxybenzoic acid is the major phenolic acid component in faba bean leaves; its mean value reached 176.4, 147.4, and 138.5 µg g^-1^ DW in Giza 716, Nubaria 5, and Nubaria 4 varieties, respectively (Table 6a-d). The level of this phenolic acid in the three tested varieties was significantly reduced by 3- 5 spray treatments as compared with the corresponding un-sprayed ones. The lowest value of this compound was recorded for three sprays in Giza 716 and for two sprays in both Nubaria varieties. Concentrations of vanillic and syringic acids in the three tested varieties showed great variations in their responses to different spray treatments and did not appear to have any unique effects. But protocatechuic acid in the three tested varieties showed a significant increase with 3–5 spray treatments. Depending on the mean value of each variety, it can be observed that Giza 716 plants accumulated more levels of *P*-hydroxybenzoic, vanillic, and syringic than those accumulated by the other tested varieties.

**Table 6:** Effect of splitting sub-lethal dose of glyphosate on phenolic acids composition in the leaves of three faba bean varieties, 7 days after treatments.

Variety							
Sprays	Giza 716		Nubaria 4	Nubaria 5	Giza 716	Nubaria 4	Nubaria 5
(i) Benzoic-derived phenolic acids (µg g^−1^ DW)							
	(a) *p*Hydroxybenzoic acid				(b) Vanillic acid		
5 Sprays	158.6^BC^		130.7^BC^	123.7^B^	51.3^B^	63.1^C^	115.9^A^
4 Sprays	168.4^B^		110.2^C^	108.0^C^	146.9^A^	129.4^AB^	103.7^B^
3 Sprays	149.2^C^		117.0^C^	123.0^B^	123.6^A^	38.1^D^	72.2^C^
2 Sprays	206.5^A^		146.8^B^	198.6^A^	61.6^B^	156.0^A^	25.6^D^
Control	199.6^A^		188.0^A^	183.8^A^	128.2^A^	106.6^B^	27.4^D^
Mean	176.5^a^		138.5^b^	147.4^b^	102.3^a^	98.6^a^	69.0^b^
	(c) Protocatechuic acid				(d) Syringic acid		
5 Sprays	53.2^A^		16.7^C^	73.2^B^	8.26^E^	16.20^B^	2.60^C^
4 Sprays	47.3^AB^		27.9^AB^	94.4^A^	45.52^A^	3.88^C^	14.02^A^
3 Sprays	43.0^B^		32.6^A^	82.9^AB^	26.73^C^	1.01^C^	9.56^B^
2 Sprays	25.0^C^		21.8^B^	45.7^C^	19.92^D^	26.03^A^	2.30^C^
Control	42.2^B^		15.1^C^	72.9^B^	38.80^B^	20.22^AB^	1.00^C^
Mean	42.14^b^		22.82^c^	73.82^a^	27.85^a^	13.47^b^	5.90^c^
(ii) Cinnamic-derived phenolic acids (µg g^-1^ DW)							
	(e) Coumaric acid				(f) Ferulic acid		
5 Sprays	77.1^AB^	103.2^B^		74.3^C^	11.50^B^	16.00^A^	12.16^AB^
4 Sprays	68.8^B^	60.7^C^		112.3^A^	15.91^AB^	14.89^B^	9.80^C^
3 Sprays	78.6^AB^	52.8^C^		79.6^BC^	11.56^B^	14.08^B^	13.96^A^
2 Sprays	82.9^A^	59.9^C^		83.6^B^	19.03^A^	16.13^A^	11.67^B^
Control	85.6^A^	145.2^A^		84.4^B^	19.67^A^	16.02^A^	11.18^B^
Mean	78.6^a^	84.4^a^		86.8^a^	15.53^a^	15.46^a^	11.75^b^

The same capital letters in a column are not significantly different (p < o.o5).

The same small letters in a row are not significantly different (p < o.o5).

(ii) Cinnamic-derived phenolic acid

The tendency of coumaric in the three tested varieties to decrease in response to different spray treatments was observed in most cases (Table 6e). Although four sprays produced the highest reduction effect on coumaric acid in Giza 716 and Nubaria 4, this treatment resulted in a great increase in the level of coumaric acid in Nubaria 5 plants. Depending on the mean coumaric value of each variety, there are not any significant variations between the three tested varieties. As for ferulic acid, among the tested varieties, ferulic acid varied in its effects by the number of sprays applied (Table 6f). Treating the three varieties with two sprays did not produce any significant effect on ferulic acid contents as compared with an un-sprayed one. Meanwhile, application of glyphosate in 3–5 sprays tended to reduce ferulic acid content in Giza 716 and Nubaria 4 varieties. In comparison with the other two varieties, Nubaria 5 had the lowest level of ferulic acid.

#### Mean effects of the number of sprays on phenolic acids composition

(i) Benzoic-derived phenolic acid

**Fig. 5:**
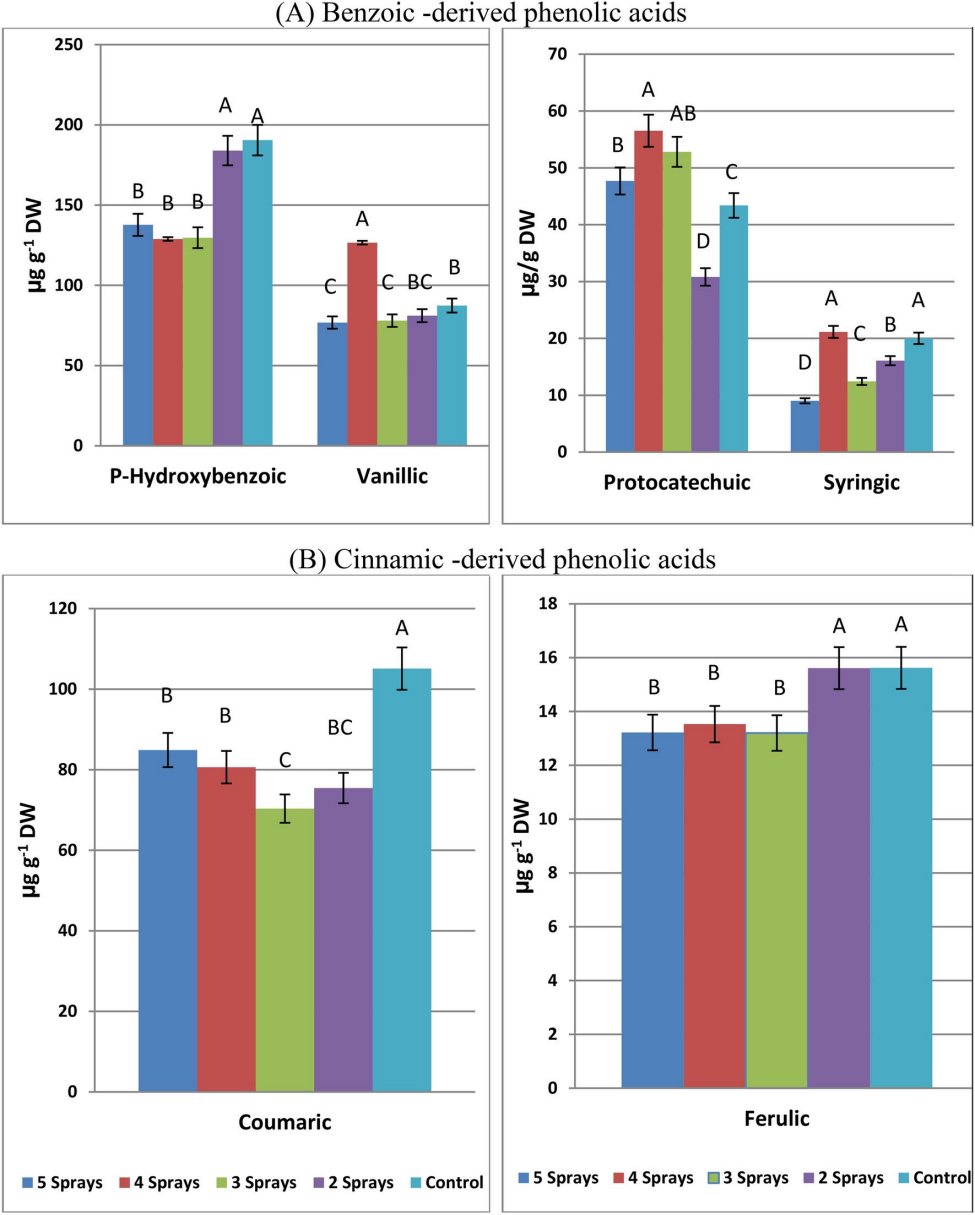
Mean effects of splitting sub-lethal dose of glyphosate on phenolic acids composition in faba bean leaves (mean value of three varieties). The same capital letters are not significantly different (p < o.o5).

Depending on the mean effect of the number of sprays, it can be observed that all spray treatments reduced *p*-hydroxybenzoic acid in faba bean leaves between 28 and 32% as compared with an un-sprayed treatment (Fig. 5A). As for vanillic and syringic acids, except for the enhancement effect of 4 sprays, levels of both compounds were significantly reduced by all sprays treatments, when compared with un-sprayed ones. On the contrary, a remarkable increase in its level was observed for 3–5 sprays treatments (Fig. 5A).

(ii) Cinnamic-derived phenolic acids

All number of sprays applied tended to reduce coumaric and ferulic acid contents as compared with the corresponding non-sprayed ones (Fig. 5B). The level of coumaric showed a decrease between 19.2% and 33.1%, as affected by all numbers of sprays, and the highest reduction effect was observed for three sprays. Also, more than 13% decreases in the level of ferulic were recorded for 3–5 spray treatments.

### Antioxidant activity

#### Effect of the number of sprays on antioxidant activity in three faba bean varieties

All glyphosate treatments caused significant decreases in antioxidant activity in the three tested varieties as compared with the corresponding control (Table 7a). The reduction ranged between 15.3% and 32.3% for Giza 716, between 11.6% and 19.5% for Nubaria 4, and between 19.6% and 42% for Nubaria 5. Among the three tested varieties, the highest mean value of antioxidant activity was observed for Giza 716.

**Table 7:** Effect of splitting sub-lethal dose of glyphosate on the levels of the antioxidant activity and activity of antioxidant enzymes in leaves of three faba bean varieties, 7 days after treatments.

Varieties							
Sprays	Giza 716	Nubaria 4	Nubaria 5		Giza 716	Nubaria 4	Nubaria 5
	(a) Antioxidant activity (µmol Trolox g^-1^ DW)				(b) Peroxidase activity (∆A/min/g FW)		
5 Sprays	746^C^	654^B^	786^B^		17.3^B^	19.6^A^	21.1^A^
4 Sprays	728^C^	604^B^	567^D^		21.8^A^	18.0^B^	18.5^B^
3 Sprays	911^B^	596^B^	673^C^		17.3^B^	16.1^C^	18.7^B^
2 Sprays	743^C^	604^B^	701^C^		15.7^B^	19.1^A^	18.1^BC^
Control	1075^A^	740^A^	978^A^		16.7^B^	12.9^D^	16.5^C^
Mean	841^a^	640^b^	741^ab^		17.8^ab^	17.1^b^	18.6^a^
	(c) Polyphenol oxidase activity (∆A/min/g FW)				(d)Superoxide dismutase activity (U/g FW)		
5 Sprays	8.20^AB^	8.35^A^		6.20^B^	15.9^B^	11.1^B^	15.0^AB^
4 Sprays	8.55^A^	8.53^A^		6.93^AB^	17.0^AB^	18.0^A^	18.0^A^
3 Sprays	8.48^A^	7.30^B^		7.70^A^	18.7^A^	19.4^A^	13.0^B^
2 Sprays	7.55^B^	8.38^A^		6.65^AB^	15.1^B^	20.5^A^	10.9^BC^
Control	7.75^B^	5.78^C^		4.43^C^	16.8^AB^	9.0^C^	7.2^C^
Mean	8.11^a^	7.67^b^		6.38^C^	16.7^a^	15.6^a^	12.8^b^

The same capital letters in a column are not significantly different (p < o.o5). The same small letters in a row are not significantly different (p < o.o5).

#### Mean effects of the number of sprays on antioxidant activity

**Fig. 6:**
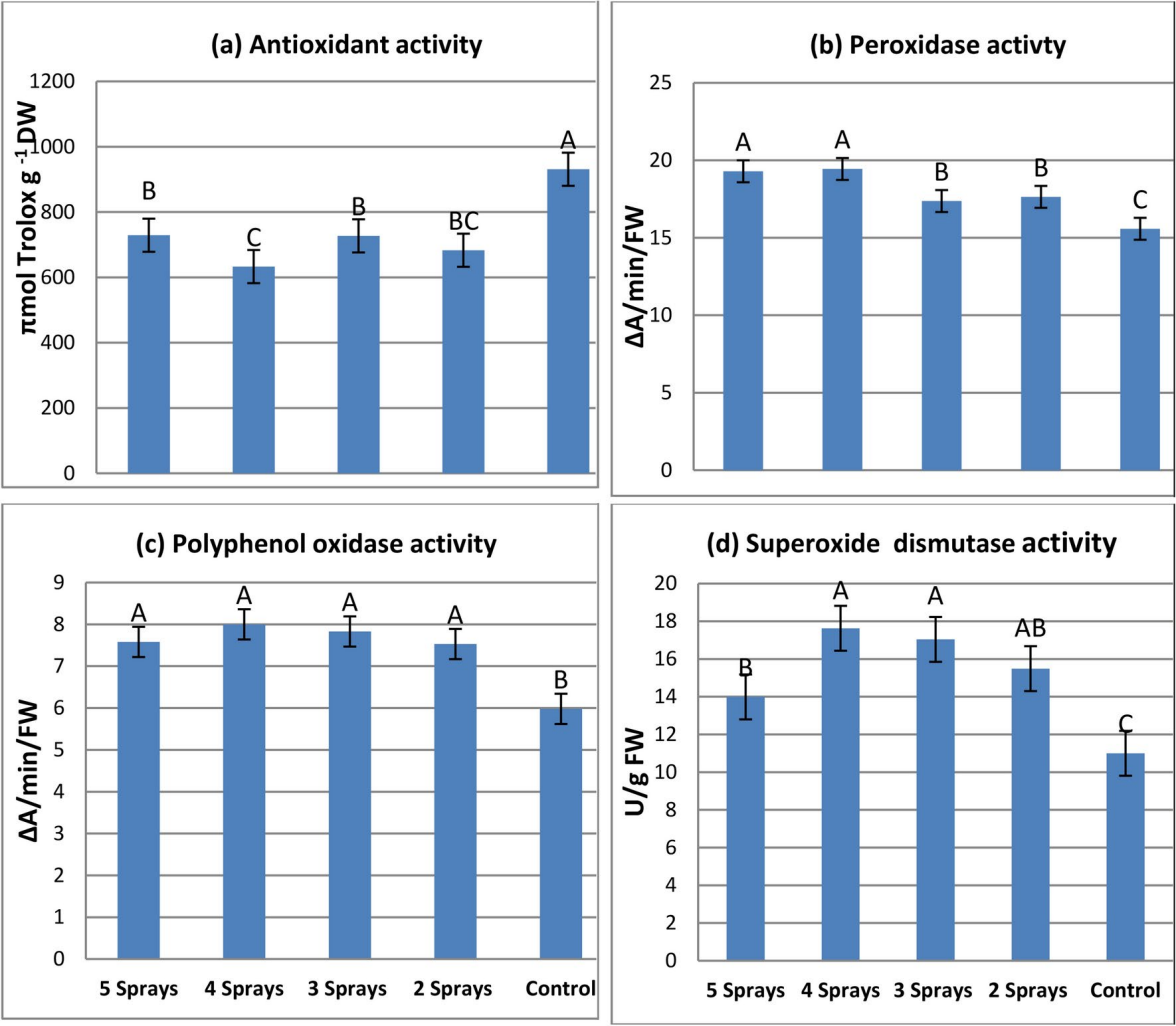
Mean effects of splitting sub-lethal dose of glyphosate on the levels of the antioxidant activity and activity of antioxidant enzymes in faba bean leaves (mean value of three varieties), 7 days after treatments. The same capital letters are not significantly different (p < o.o5).

The mean values of antioxidant activity for all spray treatments showed significant decreases when compared with those of unsprayed ones (Fig. 6a). The greatest decrease in antioxidant activity (32%) possessed 4 sprays, followed by 2 sprays (26.6%).

### Activity of antioxidant enzymes

#### Effect of the number of sprays on enzyme activities in the three faba bean varieties

A tendency of three antioxidant enzymes in the three tested varieties to increase their activities as affected by all spraying treatments was observed (Table 7b-d). The enhancement effect of spray treatments on the activity of three enzymes varied between the tested varieties. Depending on the mean value of enzyme activity for each variety, it can be observed that maximum activity of PPO and SOD was recorded for the Giza 716 variety, but maximum activity of POD was recorded for the Nubaria 5 variety.

#### The mean effects of the number of sprays on antioxidant enzyme activities

The data presented in Fig. 6b–d showed a significant increase in activity of the three antioxidant enzymes by all spray numbers as compared with un-sprayed ones. All spray treatments caused more than 13% increases in POD activity, accompanied by more than 25% increases in either PPO or SOD activities. A gradual increase in the activities of the three enzymes was observed by increasing the number of sprays from 2 to 4. In turn, the three enzymes showed their maximum activities with four sprays, which increased POD by 24.8%, PPO by 33.8%, and SOD by 60.3% relative to the corresponding un-sprayed ones.

## Discussions

Under parasite-free conditions, we examined the capability of dividing the low glyphosate dose to reduce its negative effects on plant growth, shikimate-related metabolites, and antioxidant status in three faba bean varieties. The low glyphosate dose (2.13 mM) was divided into two to five sprays and applied sequentially to the foliage of three faba bean varieties at the beginning of the flowering stage and within a period of one month. The results indicated that after 40 days from all glyphosate treatments, a cumulative depressive effect on the growth and productivity traits of the three faba bean varieties was observed during the two examined seasons. Limited studies on the effect of low glyphosate doses used to control *Orobanche* on host growth were conducted under parasite-free conditions. An inhibition effect on faba bean growth and yield due to applying glyphosate at different doses was previously observed^4,21^. In accordance with our results, a negative effect of the glyphosate spraying treatments (one, two, and three sprays at 86 g a.i. ha-1) on the growth and yield of faba bean plants was recorded^1^. Moreover, the foliar application of glyphosate at 80 g a.i. ha^-1^ applied twice on faba bean leaves reduced plant height and seed yield by 19% and 28%, respectively, as compared to the control^22^. The phytotoxic effect of glyphosate may be attributed to its inhibition effects on the shikimate pathway, which indirectly influences a variety of plant processes^23^. The present results indicated that the phytotoxicity of glyphosate split treatments differed according to faba bean varieties, examined seasons, and the number of sprays applied. Differences between the three faba bean varieties in their effects with spray treatments will reflect variations in their sensitivity to glyphosate. Variation between faba bean varieties (Misr 1, Giza 3, and Giza 843) in their growth and productivity traits as affected by glyphosate applied twice at 75 cc fed^-1^ was previously observed^24^. Current results revealed that the glyphosate-treated plants tended to produce the largest reductions in biomass and pod production in the first season when compared with the second one. Moreover, the number of sprays applied varied in their reduction effects among the two studied seasons. The maximum reduction effect was noticed for twice- sprayed treatment in the first season and for five-sprayed treatment in the second one. These results may reflect the differences in the interaction between faba bean plants and glyphosate under different weather conditions for the two examined seasons^25^. In this concern, variation in yield reductions of faba bean due to different glyphosate spray treatments across environments and year-round seasons was observed^1^. The depressing effect on faba bean growth by glyphosate split treatments manipulating that splitting the low glyphosate dose still induces inhibition effects on the EPSPS enzyme in the shikimic pathway of the treated plants^6^.

The cumulative effects of splitting glyphosate treatments on the metabolites related to the shikimate pathway and the antioxidant status of three faba bean varieties was measured after a week of spraying. As expected, a great accumulation of shikimic acid was recorded for all splitting treatments as compared with those of un-sprayed plants. The EPSPS enzyme in the shikimate pathway is the target of glyphosate herbicide and its substrate, shikimate-3-p, is rapidly hydrolyzed and accumulates as shikimic acid in glyphosate-treated plants^9^. In line with our results, spraying corn and soybeans with sub-lethal glyphosate doses (less than 230.4 g a.i. ha^-1^) increased the level of shikimic acid by up to 969% in corn and 33,000% in soybeans^26^. The great increase in shikimate contents by all spraying treatments implied that dividing the low glyphosate rate into 2–5 doses continued to induce inhibition in shikimic acid pathway as a result of the cumulative effect of glyphosate under all splitting treatments. The lesser increase in shikimate was recorded in this study for the twice-sprays treatment, proposing the potential of this treatment, at least in a part, to reduce glyphosate stress in comparison with the other number of sprays applied. Moreover, the great accumulation of shikimate observed for Nubaria 5 as affected by all spray treatments led to the conclusion that this variety is more sensitive to glyphosate than Giza 716 and Nubaria 4 varieties^6^.

Phenolic compounds are a large group of secondary metabolites in plants, including flavonoids, phenolic acids, tannins, lignin, lignans, and coumarins. These compounds are biosynthesized via the shikimic acid pathway in plants, mainly from Phe and Tyr^7^. The current results revealed that all splitting treatments tended to reduce the total phenolics and total flavonoids in the three tested varieties as compared with the corresponding control. These results are in accordance with the results of many investiigators^2,27^. Phenolic reduction might result from the cumulative inhibitory effects of all splitting treatments on the shikimate pathway^9^. The greatest cumulative inhibition effect was observed on total phenolics for 4 sprays and on total flavonoids for 4–5 sprays. The results indicated that tested faba bean varieties varied in their phenolic contents^28^. Moreover, these varieties varied in their response to different splitting treatments, supposing a difference in their sensitivities to glyphosate treatments^29^.

The detectable benzoate-derived phenolic acids included *p*-hydroxy benzoic acid, vanillic acid, protocatechuic acid, and syringic acid, while coumaric acid and ferulic acid were the only cinnamic-derived phenolic acids that were detected in faba bean leaves. The tendency of most phenolic acids to decrease as affected by glyphosate treatments is in accordance with the previously mentioned decrease in total phenolics by spray treatments^30^. But the tendency of protocatechuic acid to accumulate as affected by different splitting treatments in this study is in general consistent with those obtained by Zulet-Gonzalez et al.^27^. In accordance with these results, exposing velvetleaf plants to 5 mM glyphosate resulted in decreases in the phenolic acid composition, but a 12-fold increase in protocatechuic acid was observed^31^. Differences in the response of phenolic acids to glyphosate treatments led to the hypothesis that these treatments may activate an alternative pathway not regulated by PAL enzyme activity^32^. The three tested varieties did not show any homogenous pattern in their phenolic acid contents in response to different sprays treatments, with different patterns for each phenolic acid depending on the tested varieties and number of sprays. In this regard, the phenolic acid composition showed significant variations between faba bean varieties^29^. In the current study, the low glyphosate dose was applied as three sprays tended to produce the greatest decrease in the levels of *p*-hydroxybenzoic, vanillic, syringic, coumaric, and ferulic acids.

The three AAAs (Phe, Tyr and Trp) are formed from chorismate, the end product of the shikimate pathway^7^; theoretically, the inhibition of this pathway by glyphosate prevents the biosynthesis of these amino acids^6^. Differential effects of glyphosate on free AAAs were previously observed by many investigators^5,33^. Current results indicated that all splitting treatments caused increases in free Phe and Trp as well as in Tyr in most cases. In accordance with the obtained results, the accumulation of free AAAs by glyphosate was previously reported^34–36^. It was proposed that this increase in AAAs might be due to the increase in protein turnover, either for a higher breakdown rate or a lower biosynthesis rate^30^. In this regard, the application of glyphosate resulted in increases in protein turnover, which induce an increase in the free aromatic and non-aromatic amino acids^27^. Among the three tested varieties, we found that spray treatments did not show any homogenous pattern in their effects on the three AAAs. Since each AAA showed different patterns depending on the tested variety, and number of sprays applied.

Oxidative stress can occur in response to environmental stress such as herbicides, leading to an increase in the levels of the ROS^37^. Decreases in antioxidant activity by all glyphosate treatments in the present study confirm those observed in the previous study^12^. Decreases in antioxidant activity in glyphosate treated plants can occur due to the inhabitation of the antioxidant compounds that are biosynthesized through the shikimate pathway or due to the enhancement effect of glyphosate on the oxidation of biological compounds and producing more ROS^2,38^. The decrease in the phenolic contents and antioxidant activity after exposure to different numbers of glyphosate sprays is in general agreement with previously reported results^2,39^. Since phenolic compounds are considered to be major non-enzymatic antioxidant compounds that are capable of quenching ROS^40^. The levels of the total phenolic content may estimate the antioxidant potential of plants and their herbicide tolerance^41^. These results led to suggestion that the reduction in antioxidant activity by different numbers of spray applied would be directly related to total phenolic inhibition by glyphosate treatments^12^.

The antioxidant enzymes are responsible for scavenging the toxic ROS and protecting plants from the oxidative stress^17^. In the current study, the activities of the three antioxidant enzymes, i.e., POD, PPO, and SOD, showed significant increases as affected by all spray treatments. Such increases in antioxidant enzymatic activities may help to degrade ROS, regulate lipid peroxidation, and avoid plants from glyphosate oxidative stress^17^. The obtained results indicated that among the tested varieties, the activities of antioxidant enzymes varied in their effects by different splitting treatments, and the largest changes in the antioxidant status were recorded for the four sprays treatment. This treatment caused the highest decrease in antioxidant activity and phenolic contents, accompanied by the largest increase in antioxidant enzyme activities in attempt of treated plants to cope with glyphosate stress^42^.

Generally, there is a lack of information in the literature on the effect of the low glyphosate dose on growth and herbicide target metabolites of plants that are grown under parasite-free conditions. The novelty results of this study indicated that splitting the low glyphosate dose (170 g a.i. ha^-1^) into 2–5 sprays continued to exert cumulative toxic effects on growth and glyphosate target metabolites in faba bean plants. These results appear to disagree with Goldwasser et al.'s results^13^. Who found that splitting the low glyphosate dose in order to control the *Orobanche* parasite can reduce glyphosate-induced phytotoxicity to host plants while still sustaining the toxic effect on the attached parasite? The conflicting results may be due to the fact that Goldwasser et al.'s study was conducted under parasitic conditions without determining the effect of splitting treatments on the target herbicide metabolites.

## Conclusions

Dividing the sub-lethal glyphosate dose into two-five portions tended to produce cumulative negative effects on faba bean growth and the composition of the metabolites related to the shikimic acid pathway and antioxidant status. The inhibition effects varied depending on the number of sprays, variety, and season. The highest reduction effect on faba bean growth was observed by dividing the glyphosate dose into two sprays in the first season and into five sprays in the second one. Glyphosate splitting treatments enhanced shikimate, protocatechuate, and Phe in the three faba bean varieties. These treatments reduced levels of total phenolics, total flavonoids, and the most detected phenolic acids. The oxidative stress of all splitting treatments was evidenced by a reduction in phenolic content and antioxidant activity, accompanied by the induction of superoxide dismutase, peroxidase, and polyphenol oxidase activities. The results concluded that splitting the low glyphosate dose continued to exert cumulative toxic effects on growth and glyphosate target metabolites in faba bean plants.

## Data Availability

All data generated or analysed during this study are included in this published article.
